# Image datasets of cocoa beans for taxonomy nuances evaluation

**DOI:** 10.1016/j.dib.2019.104655

**Published:** 2019-10-15

**Authors:** F.A. Santos, E.S. Palmeira, G.Q. Jesus

**Affiliations:** Universidade Estadual de Santa Cruz, Brazil

**Keywords:** Cocoa beans, Cut Test, Object positioning, Taxonomy evaluation

## Abstract

There are some classification methods that generate nuances in the final accuracy caused by objects positioning, framing and damage. These occurrences may result in a drop of accuracy in computer vision systems that were trained with structured static datasets and are intended to be used in day-to-day applications in which the images are not always as organized as the trained dataset, like some biometric classification systems such as iris and fingerprint. In this regard, this paper presents six image datasets processed with different methods to help researchers analyze the impact of object positioning, framing and damage in their taxonomies.

**Table:** Specifications Table

Subject area	*Agronomy, Image Processing*
More specific subject area	*Crop Science*
Type of data	*Images (JPG)*
How data was acquired	*Different image processing methods, implemented with R language and MATLAB, were applied to an existing dataset of cut-test-classified cocoa beans.*
Data format	*Raw (preprocessed .JPG image files.)*
Experimental factors	*The methods were selected with the intent of generating datasets to facilitate the use and comparison of different classification methods and of the impact of the positioning of objects in images.*
Experimental features	*The source dataset (presented in*Ref. [1]*) was submitted to multiple research groups in Brazil and some tests (such as with fuzzy associative memories) returned different accuracies for the same objects as their position were algorithmically manipulated* [2,3].
Data source location	*Ilhéus, Bahia, Brazil.*
Data accessibility	*Data is available at Mendeley Data, under the doi: <**https://doi.org/10.17632/pcx7mj68yn.4**> (also in Ref.[**4**]).*
Related research article	*Felipe A. Santos (2019).“Modelagem de Um Sistema de Visão Computacional para a Classificação de Amêndoas de Cacau na Prova de Corte (Master's thesis)”. State University of Santa Cruz, Ilhéus, Brazil*.

**Table:** 

**Value of the Data** • These data can be used to test the impact of the objects positioning in the computational classification methods; • Those datasets can facilitate the comparison between the methods accuracies of different research groups; • The data will allow the creation, training and improvement of computational vision models to help specialist in the Cut Test classification; • The data can be used to evaluate the impact of different damage levels caused to images during their processing regarding their classification methods results.

## 1 Data

**Fig. 1:**
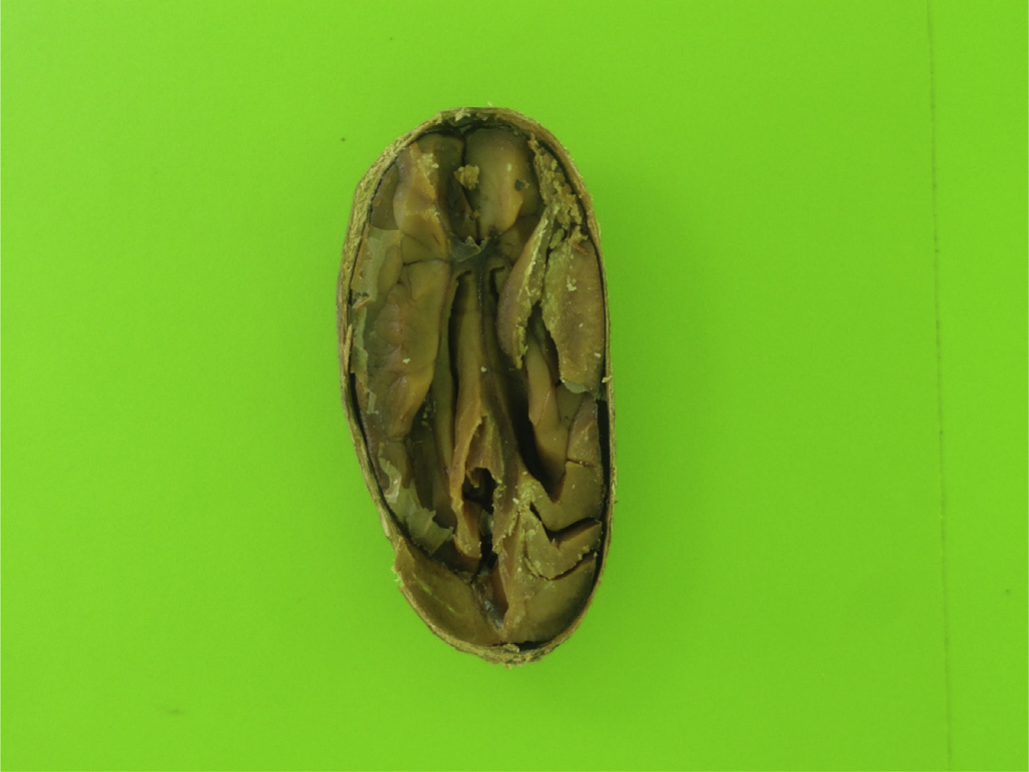
A Compartmentalized Brown bean from the source dataset.

**Fig. 2:**
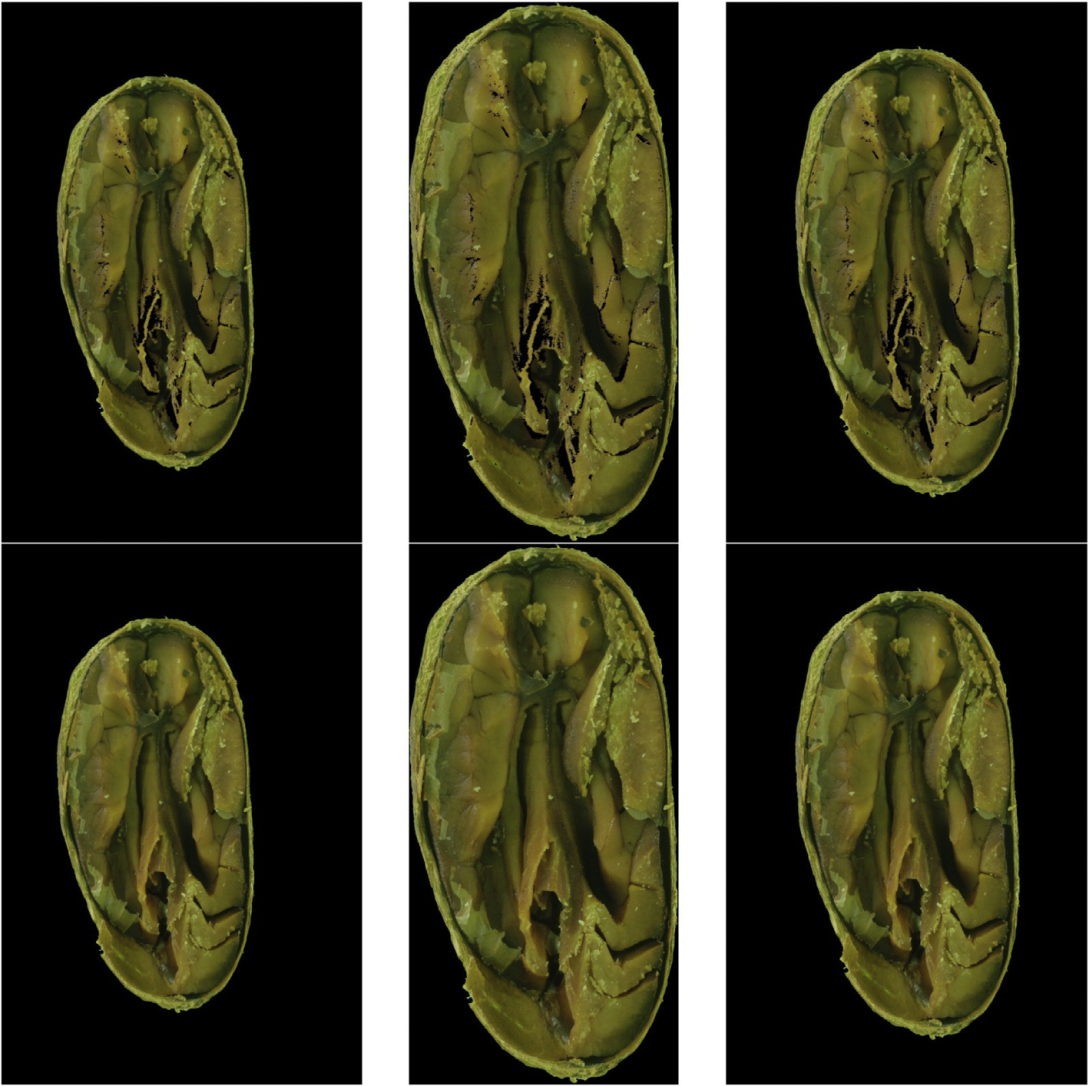
The six versions created from the bean from Fig. 1.

Six image datasets of cut-test-classified cocoa beans were created from the research presented in Ref. [3]. Each dataset contains 14 classes (namely: Compartmentalized Brown, Compartmentalized White, Compartmentalized Partially Purple, Compartmentalized Purple, Compartmentalized Slatty, Plated Brown, Plated White, Plated Partially Purple, Plated Purple, Plated Slatty, Moldered, Flattened, Brittle and Agglutinated) with 100 images per class, totaling 1400 images per dataset. Fig. 1 presents an image of the source dataset and Fig. 2 presents the six results of the same beans accordingly with the preprocessing methods.

## 2 Materials and methods

This section brings the explanation about the processing applied to the source image to produce the resulting datasets. Each section of this chapter will present the methods used to reach each of the six preprocessed versions. Those methods were obtained through empirical tests with a fuzzy associative memories implementation sensible to objects positioning, those six datasets versions presented six different accuracies for the same classification method, with a standard deviation of approximately 10.07% between them.

### 2.1 A-Method: Background Removed

**Fig. 3:**
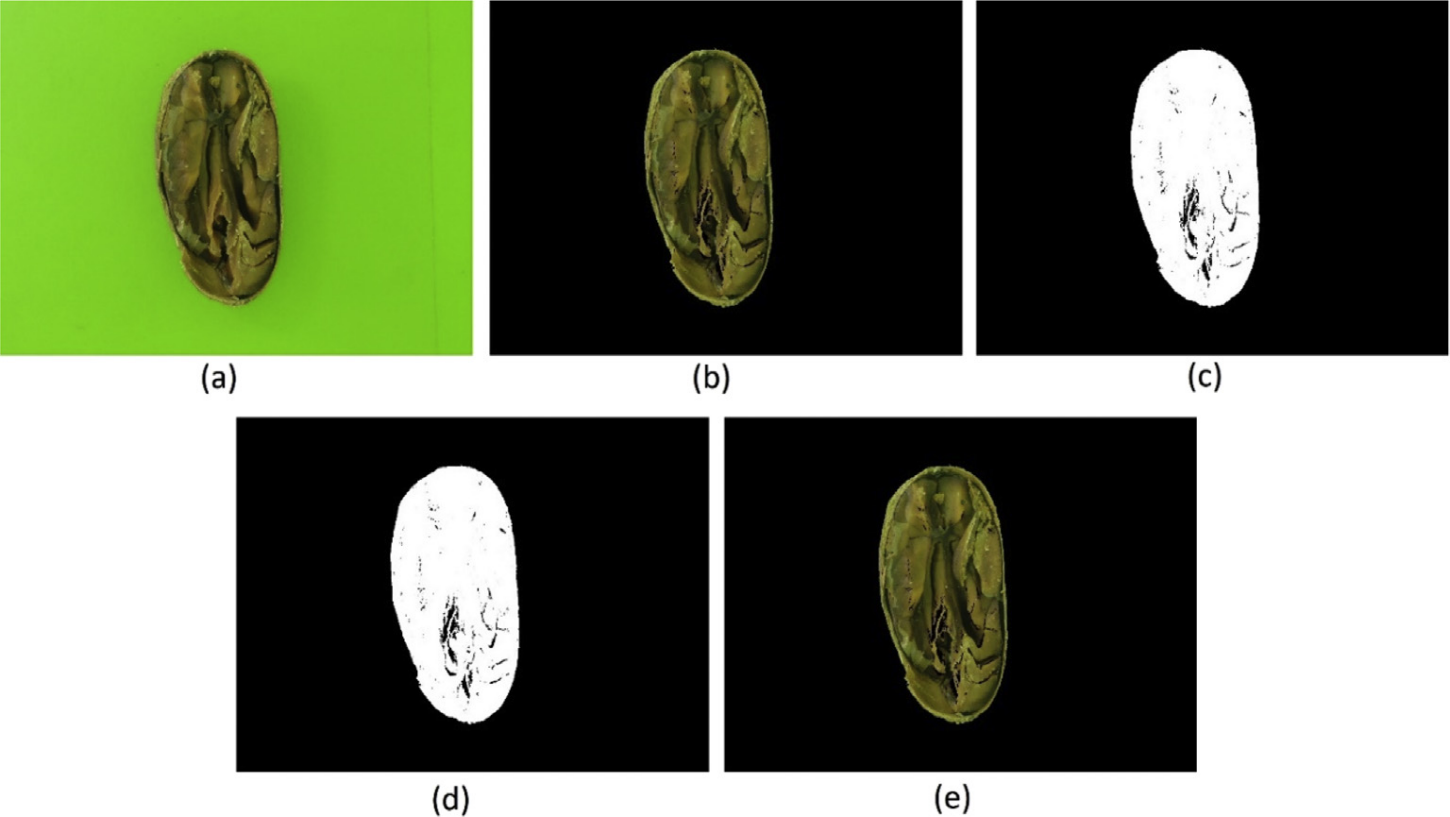
(a) Source; (b) Background removed; (c) Binarized; (d) Preservation of largest body; (e) Application of mask in source image.

Presented under the name of “background_removed_-_version_1_-_method_a.rar” in the repository, five steps (see Fig. 3) were applied to generate the images of this dataset, being:

i. The CIELAB color space were used to remove the background;
ii. The image was binarized, everything that was not pure RGB black were turned into pure RGB white;
iii. All connect white pixels were labeled as regions and only the largest region of the step (ii) were preserved;
iv. The (iii) result were applied as a mask in the source image.

### 2.2 A-Method: Only image

**Fig. 4:**
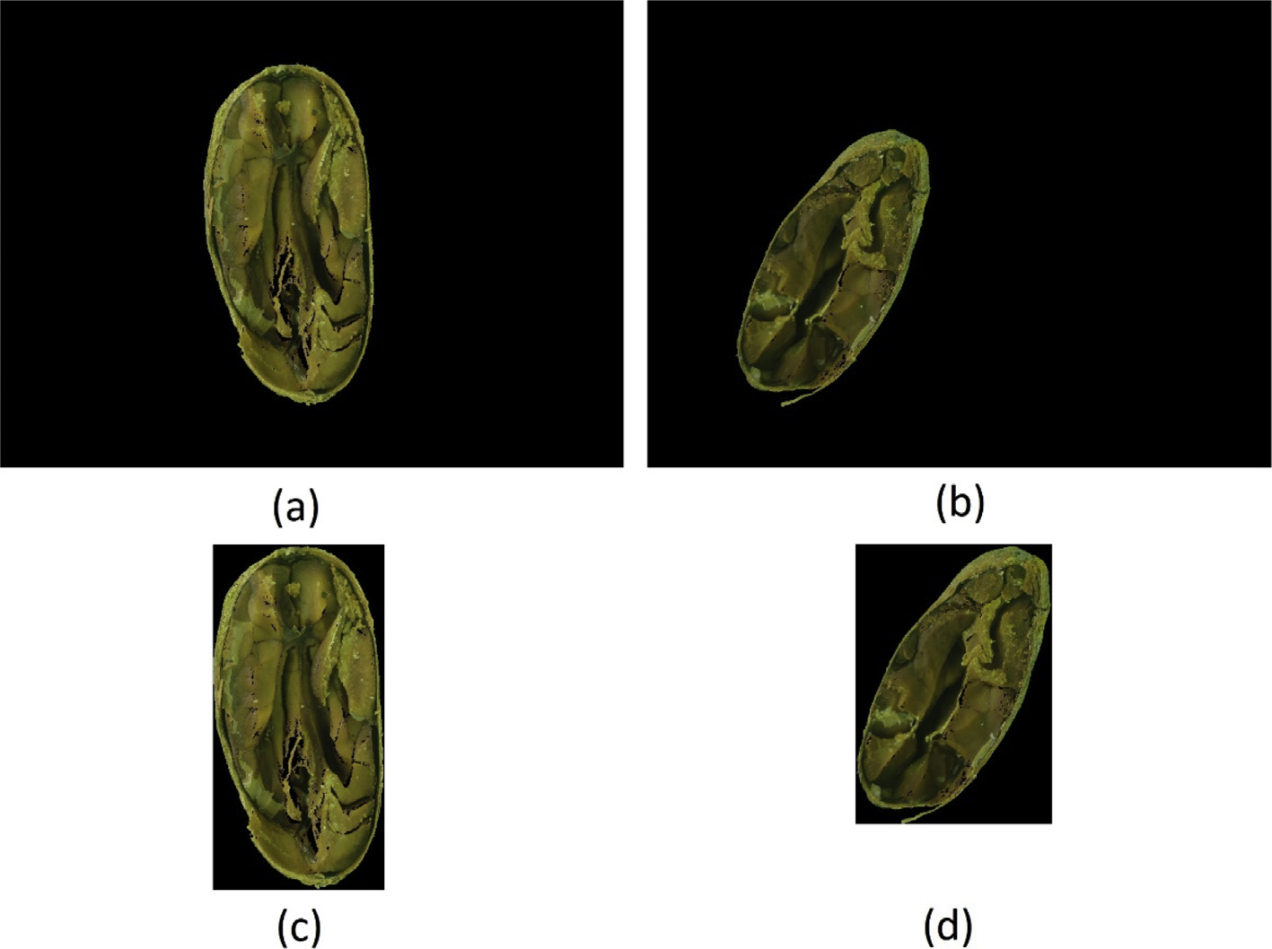
(a) and (b) are images from the “A-Method: Background Removed” and (c) and (d) are their respective cropped images to the minimum fitting rectangle.

To create this dataset all images resulted from the “ A-Method: Background Removed ” were cropped to the minimum rectangle capable of fitting each bean, resulting in images with varying widths and heights, as shown in Fig. 4. This dataset is under the name “framed_and_centralized_-_version_1_-_method_a.rar” in the repository.

### 2.3 A-Method: Framed and centralized

**Fig. 5:**
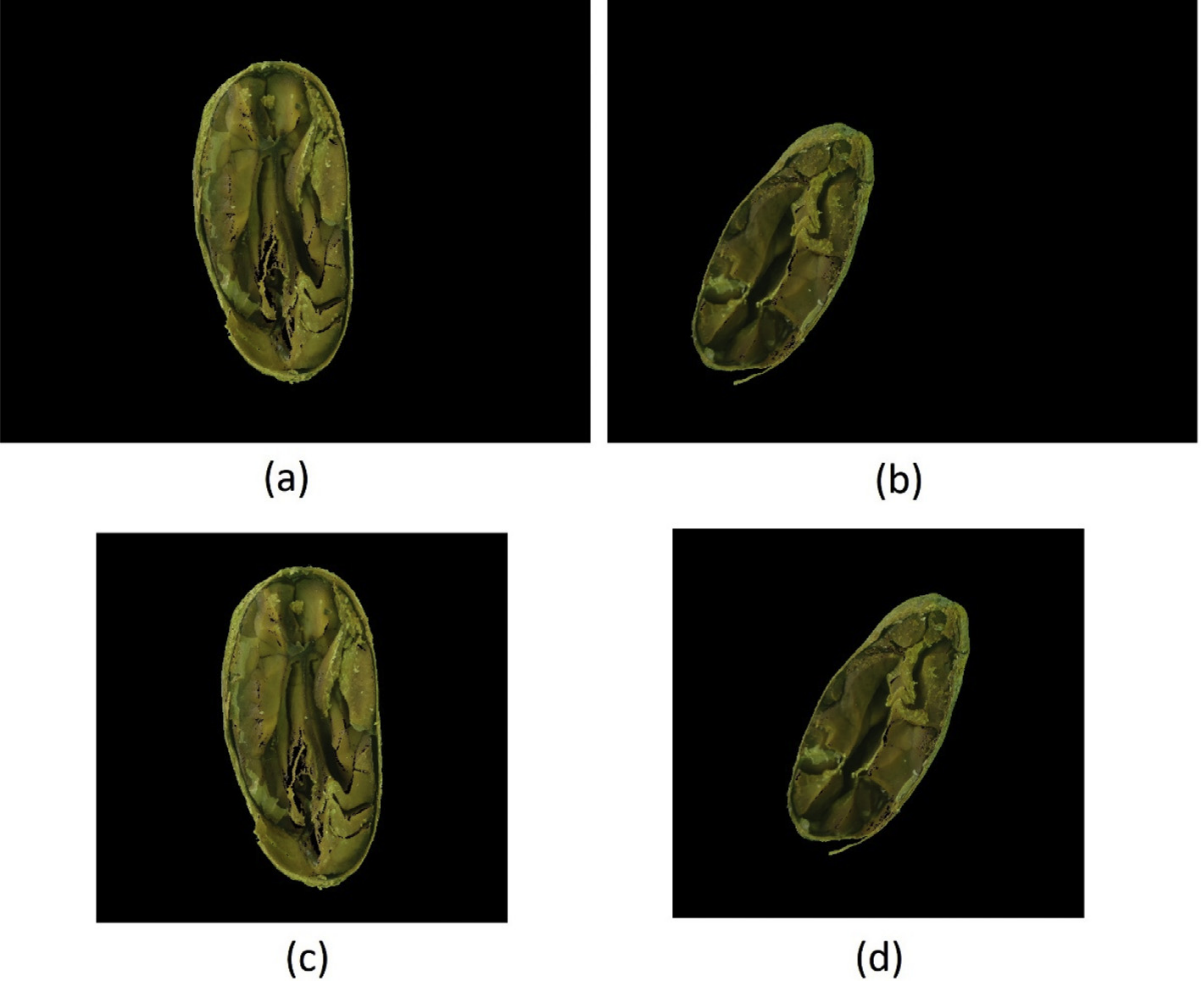
(a) and (b) are images from “A-Method: Background Removed” and (c) and (d) are their respective centralized beans in the created frame.

All beans from “ A-Method: Background Removed ” were measured and then centralized in the smallest rectangle capable of fitting all beans, thus all images have the same dimensions: 3011x2851. Two samples of this process can be seen in Fig. 5. This dataset is under the name “framed_and_centralized_-_version_2_-_method_a.rar” in the repository.

### 2.4 B-Method: Background Removed

**Fig. 6:**
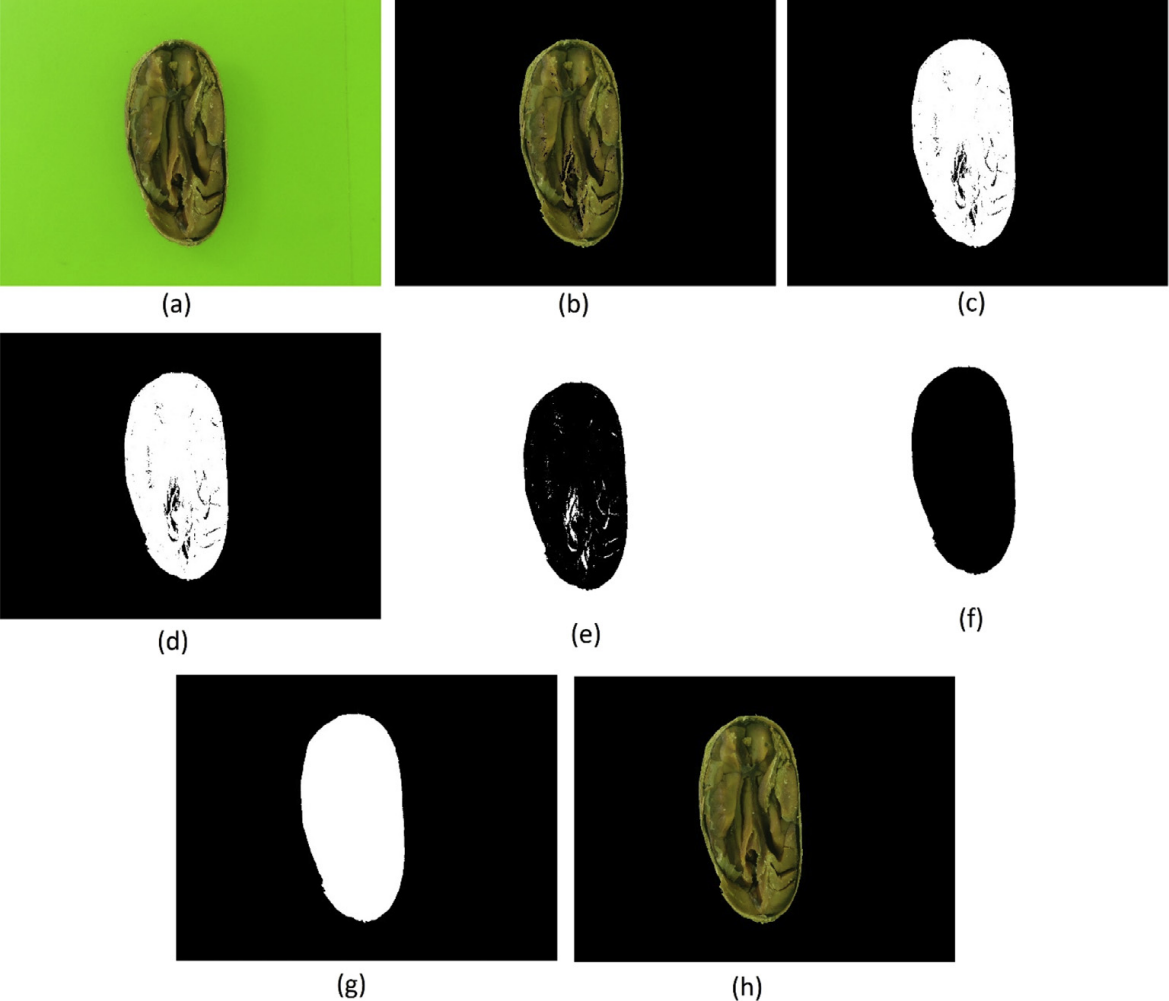
(a) Source; (b) Background removed; (c) Binarized; (d) Preservation of largest body; (e) Inverted; (f) Preservation of largest body; (g) Inverted; (h) Application of mask in source image.

This dataset (named “ background_removed_-_version_2_-_method_b.rar ” in the repository) was created with additional steps to restore the damage caused to some beans during the background removal, as shown in Fig. 6, being:

i. The CIELAB color space were used to remove the background;
ii. The image was binarized, everything that was not pure RGB black were turned into pure RGB white;
iii. All connect white pixels were labeled as regions and only the largest region of the step (ii) were preserved;
iv. The image was inverted;
v. All connect white pixels were labeled as regions and only the largest region of the step (iv) were preserved;
vi. The image was inverted again;
vii. The (vi) result were applied as a mask in the source image.

**Fig. 7:**
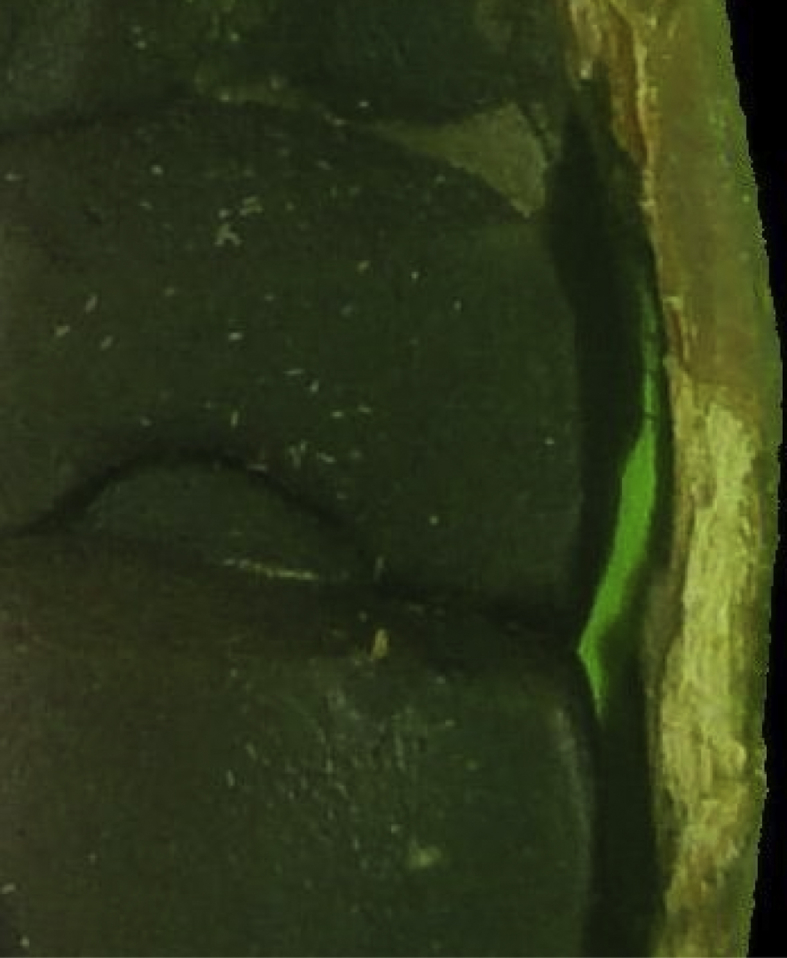
Sample of restored background.

Important to perceive that the process to restore damages to the beans also restore parts of the backgrounds that were contained inside them, as shown in Fig. 7, caused by hollow areas (such as broken ones) in the beans.

### 2.5 B-Method: Only beam

**Fig. 8:**
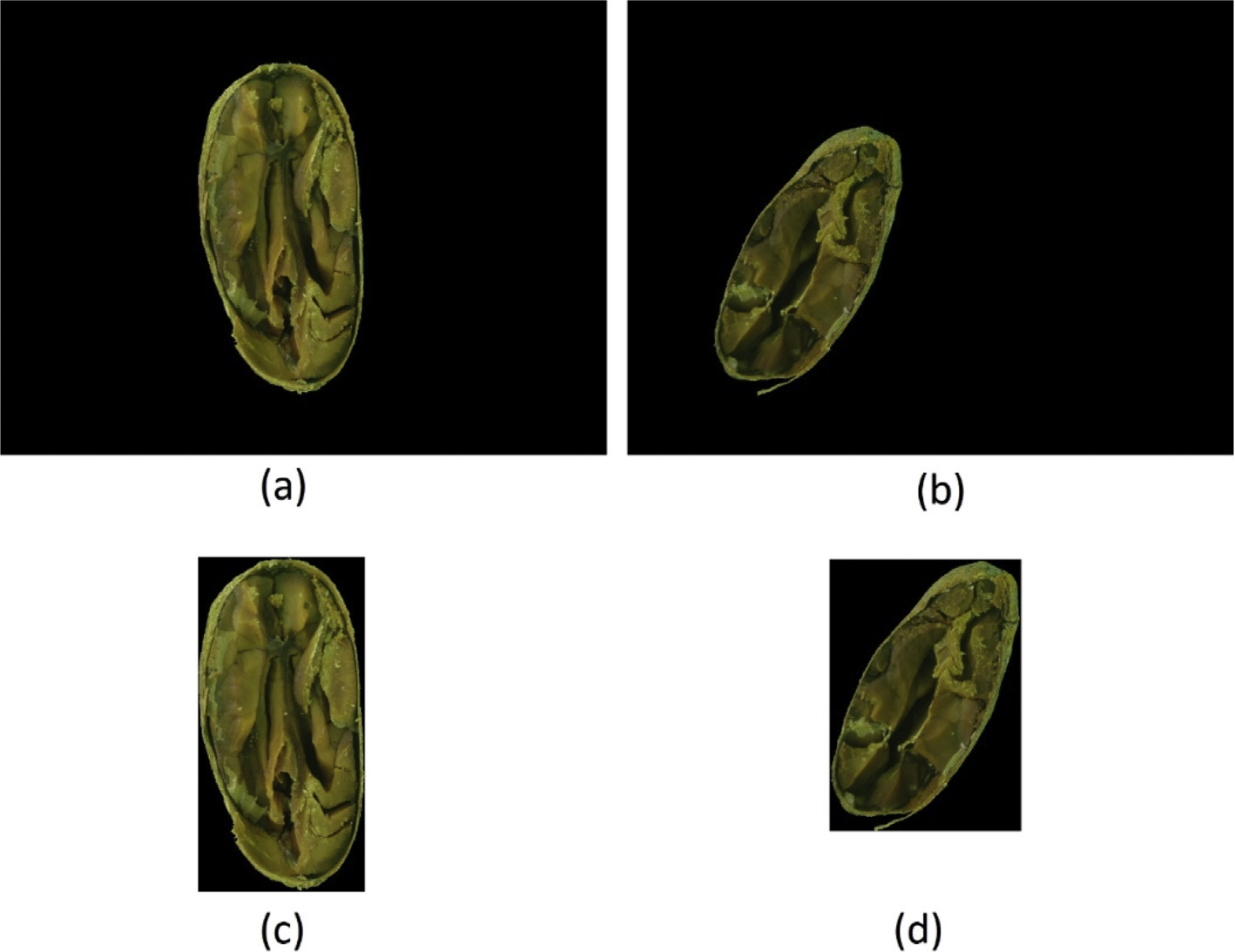
(a) and (b) are images from the “B-Method: Background Removed” and (c) and (d) are their respective cropped images to the minimum fitting rectangle.

The same process to create “ A-Method: Only Image ” were applied to the “ B-Method: Background Removed ” to create this dataset (see Fig. 8). This dataset is under the name “framed_and_centralized_-_version_3_-_method_b.rar” in the repository.

### 2.6 B-Method: Framed and centralized

**Fig. 9:**
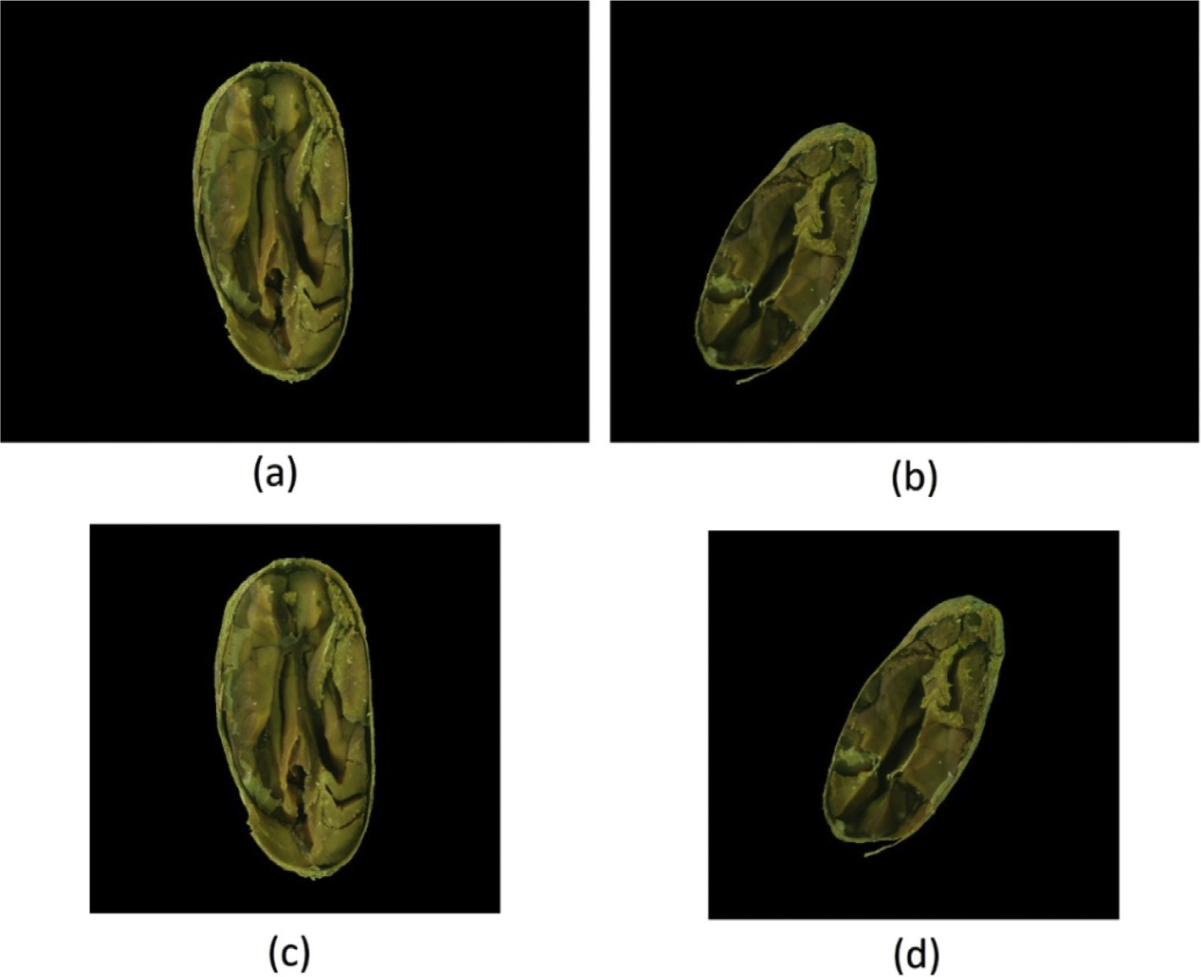
(a) and (b) are images from “B-Method: Background Removed” and (c) and (d) are their respective centralized beans in the created frame.

The same process to create “ A-Method: Framed and Centralized ” were applied to the “ B-Method: Background Removed ” images to create this dataset. The rectangle obtained were of the same dimensions as the one from “ A-Method: framed and centralized ” (3011x2851). Two samples of this process can be seen in Fig. 9. This dataset in under the name “framed_and_centralized_-_version_4_-_method_b.rar” in the repository.
